# A survey of the availability in Canadian pharmacy chains of over-the-counter natural health products for menopause symptoms

**DOI:** 10.1186/s12906-015-0608-5

**Published:** 2015-03-27

**Authors:** Jennifer Croden, Sue Ross, Nese Yuksel, Beate C Sydora

**Affiliations:** Department of Obstetrics and Gynecology, Faculty of Medicine and Dentistry, University of Alberta, 5S131 Lois Hole Hospital for Women, Robbins Pavilion, Royal Alexandra Hospital, 10240 Kingsway Avenue NW, Edmonton, AB T5H 3 V9 Canada; Faculty of Pharmacy and Pharmaceutical Sciences, 3-171 Edmonton Clinic Health Academy, 11405 – 87 Avenue NW, Edmonton, AB T6G 1C9 Canada

**Keywords:** Over-the counter products, Natural health products, Menopause, Menopause symptoms, Women’s choice

## Abstract

**Background:**

Menopause is a natural phase in a woman’s aging process, characterized by the cessation of menstruation. Women who are going through the menopause transition can experience physiological symptoms that significantly impact their quality of life. Concern about adverse effects of traditional hormone therapy often leads women to purchase over-the-counter (OTC) natural health products (NHPs). The goal of this study was toinvestigate the range of OTC NHPs for menopause available to Canadian women, and the packaging information they can access to make self-management decisions.

**Methods:**

Edmonton stores belonging to each of nine Canadian pharmacy chains were visited to identify NHPs marketed for the relief of menopausal symptoms. Details were extracted from the packaging: a) product name and manufacturer, b) Health Canada license number, c) medically active ingredients, d) claims of efficacy, e) contra-indications and warnings, and f) daily cost. Data were entered and analyzed using Microsoft Excel.

**Results:**

We identified 20 OTC NHP menopausal products, 19 of which had Health Canada license numbers. Twenty-eight medically active ingredients were identified, with the most common being black cohosh (in 14 products) and soy isoflavones (n = 7), chaste tree (n = 5), and dong quai (n = 3). Most products claimed they would relieve vasomotor symptoms, including hot flashes (n = 14) and night sweats (n = 10). Each product had a labeled contraindication for at least one specific condition. Costs per recommended daily dose ranged from $0.07 to a maximum of $2.50 (CAD$).

**Conclusion:**

Natural health products for menopausal symptoms are easily available to Canadian women. The lack of clear evidence of product efficacy makes the need for easily accessible, balanced information on this topic important for women to make well informed choices.

## Background

Menopause is a natural phase in a woman’s aging process characterized by the cessation of menstruation. The majority of women experience physiological symptoms that significantly impact their quality of life. The most common symptoms reported are hot flashes and night sweats, with 50-85% of perimenopausal women experiencing vasomotor instability [1]. Depressed mood, urogenital changes, and sleep disturbances are other symptoms commonly experienced by women during menopause [2]. While traditional hormone therapy has been found to be approximately 90% effective in reducing vasomotor symptoms [3], results of clinical trials implicating the therapy in the increased risk of cardiac events, strokes, and breast cancer [4-6] have caused women to seek alternative remedies for menopausal symptoms. Women wish to be actively involved in their choice of alternative treatments [7]. Canadian surveys suggest that as many as 60 to 90% of women may consider taking complementary and/or alternative medicine for menopause symptoms [8,9], but are concerned about obtaining credible advice and the cost of alternative treatments [7].

Natural health products (NHPs) licensed by Health Canada for sale over-the-counter (OTC) without a prescription include vitamins, minerals, herbal remedies, and homeopathic medicines. All NHPs sold in Canada are required to have a Health Canada product license (designated by an eight-digit Natural Product Number (NPN)), which indicates that safety, quality, and efficacy evidence has been assessed [10,11]. For Health Canada licensing of NHPs, efficacy evidence is required in proportional to the risk inherent in the treated condition; for example, a NHP for treating a high risk condition (one that is life-threatening without effective treatment) requires strong, corroborated evidence of efficacy, while a NHP for a low risk condition (a minor condition that may resolve itself over time) requires less efficacy evidence [10,11]. Health Canada currently approves 448 products for menopause sale consisting of single and multiple active ingredients. While there have been studies into the effectiveness of different NHPs for treating menopausal symptoms, opinions over the efficacy of these products remains mixed [12-17].

Natural health products are widely available for purchase in pharmacies across Canada, and pharmacies are considered by women as a significant resource to support decision-making [7]. Our research, a survey of NHPs sold in Canadian pharmacies, set out to identify both the range of OTC products that are available for women who are seeking non-hormonal alternatives for relief of their menopause symptoms, and the information available on product packaging to help them make their self-management decisions.

## Methods

Our descriptive study was a survey of OTC NHPs that are available to woman seeking OTC relief for menopausal symptoms in Canada-wide pharmacies in a single city, Edmonton.

### Pharmacy selection

We included nine pharmacy chains with retail stores in Edmonton, Alberta, selected to include a range of retail types (specialized pharmacy chain, grocery or department stores with in-house pharmacies). Only chains with retail stores in four or more Canadian provinces or territories were included in our study. In June 2014, one location for each of the nine pharmacy chains was visited in order to provide a snapshot of the available NHPs for menopause. The identity and availability of relevant products was initially documented in-store in June 2014.

### Product selection

Natural health products and OTC remedies were included if the product information found on the packaging stated that its recommended use was for menopause symptoms (solely or among others). Products that claimed relief for other symptoms that may be seen in menopause (i.e. sleep, anxiety) but did not specifically mention “menopause” were excluded. The name, manufacturer and NPN for each product were noted. One container of each product identified was purchased.

### Data extraction

The following data items that women might consider when selecting a NHP were collected from the packaging for each individual product:

- Name and quantity of each active ingredient in the product
- Recommended indications of the product
- Contraindications and warnings of the product
- Recommended daily dose for each product
- Cost per package (CAD$)

### Data analysis

All data were entered into a Microsoft Excel spreadsheet to categorize product and ingredient data.

Cost per recommended daily dose was calculated for each product for each store where it was identified by dividing the retail price by the number of tablets or capsules sold and multiplying by the average recommended daily dose as stated on the product’s package. The range of costs per daily dose is presented for products sold in more than one store at different retail prices.

Excel’s statistical analysis tools were used to calculate the range, mean, and standard deviation of numerical data as necessary.

## Results

### Natural health products

In the nine selected Canadian pharmacy chains we identified and purchased a total of 20 OTC NHPs available for relief of menopausal symptoms between 13 October and 18 November 2014. The median number of products available per pharmacy was eight, with a range of two to 12 products. Details of the individual products are described in Tables 1 and 2.

**Table 1:** **Details of the product, active ingredients, daily dose and cost per dose**

**a. Products with multiple active ingredients**				
**Product name/Manufacturer**	**Health Canada Licence Number**	**Ingredients active for menopause symptoms, amount per tablet 5 most common active ingredients in bold**	**Recommended daily dose (# of tablets, frequency)**	**Range of cost of average daily recommended dose** ^**a**^ **(CAD$)**
Swiss Natural HRT® Regular/Valeant Canada LP/S.E.C.	80038543	**Black cohosh** (30 mg), **chaste tree** (100 mg), **soy isoflavones** (70 mg), wild yam (50 mg), burdock (50 mg)	1, twice daily	$0.36-$0.93
Swiss Natural HRT® Extra Strength/Valeant Canada LP/S.E.C.	80019270	**Black cohosh** (100 mg), **chaste tree** (200 mg), **soy isoflavones** (100 mg), wild yam (50 mg), burdock (100 mg)	1, daily	$0.18-$0.47
Swiss Natural HRT® Nightime/Valeant Canada LP/S.E.C.	80002152	**Black cohosh** (100 mg), lemon balm (300 mg), passion flower (150 mg)	1, at night	$0.36-$0.47
Estroven®/i-Health, Inc.	80024706	**Black cohosh** (40 mg), **soy isoflavones** (37.5 mg), calcium (150 mg), folic acid (400 μg), kudzu (100 mg), magnolia (15 mg), vitamin E (30 IU), thiamin (2 mg), riboflavin (2 mg), niacinamide (20 mg), vitamin B6 (10 mg), vitamin B12 (6 μg), selenium (70 μg), boron glycinate (1.5 mg), Chinese-date seed (120 mg)	1, daily	$0.61-$0.86
Estroven® Maximum Strength/i-Health, Inc.	80032569	**Black cohosh** (80 mg), calcium (138 mg), **chaste tree** (75 mg), folic acid (400 μg), green tea (60 mg), **soy isoflavones** (102.5 mg), vitamin D3 (25 μg), vitamin E (30 IU), thiamin (5 mg), riboflavin (5 mg), niacinamide (20 mg), vitamin B6 (20 mg), vitamin B12 (25 μg), selenium (70 μg), boron glycinate (1.5 mg), chromium (120 μg), L-theanine (50 mg), caffeine (49 mg), cranberry (25 mg)	1, daily	$1.00-$1.17
Life Brand™ Menopause Relief/GFR Pharma Ltd.	Not available	**Black cohosh** (20 mg), **red clover isoflavones** (42 mg), **soy isoflavones** (425 mg)	1, daily	$0.33
femMED® Menopause Relief/femMED Formulas Inc.	80025767	**Black cohosh** (40 mg), **dong quai** (125 mg), pomegranate (75 mg), damiana (75 mg), partridge berry (75 mg), motherwort (50 mg), schizandra (50 mg)	1, twice daily	$0.70-$1.10
webber naturals® FemmeCalm Menopause Formula/WN Pharmaceuticals Ltd.	80046012	**Black cohosh** (80 mg), **chaste tree** (80 mg), **dong quai** (100 mg), gamma-oryzanol (75 mg), hesperidin (30 mg)	1, three times daily	$0.66
MENOsmart™ Plus/Lorna Vanderhaeghe Health Solutions, Inc.	80025898	**Black cohosh** (80 mg), **chaste tree** (80 mg), **dong quai** (100 mg), gamma-oryzanol (75 mg), hesperidin (75 mg), sage (75 mg)	2, morning and night	$1.10
**b. Products with single active ingredient**				
**Product name/Manufacturer**	**Health Canada Licence Number**	**Ingredients active for menopause symptoms, amount per tablet**	**Recommended daily dose (# of tablets, frequency)**	**Range of cost of average daily recommended dose** ^**a**^ **(CAD$)**
up&up™ Black Cohosh/WN Pharmaceuticals Ltd.	80007421^b^	Black cohosh (40 mg)	1-2, daily	$0.24
London *Naturals*® Black Cohosh/WN Pharmaceuticals Ltd.	80007421^b^	Black cohosh (40 mg)	1-2, daily	$0.27
equate® Black Cohosh/Vita Health Products Inc.	02238871	Black cohosh (40 mg)	1, daily	$0.12
Holista® Black Cohosh/Holista Health (Canada) Inc.	80010287	Black cohosh (40 mg)	1-2, daily	$0.07-$0.09
Be.better™ Black Cohosh/WN Pharmaceuticals Ltd.	80007421^b^	Black cohosh (40 mg)	1-2, daily	$0.29
Promensil® Regular Strength/PharmaCare Laboratories	80015467	Red clover isoflavones (40 mg)	1, daily	$0.67-$1.10
Promensil® Double Strength/PharmaCare Laboratories	80016071	Red clover isoflavones (80 mg)	1, daily	$1.23-$1.33
webber naturals® Soy Isoflavone Complex/WN Pharmaceuticals Ltd.	80015647	Soy isoflavones (50 mg)	7, daily	$0.77
Menoconfort Forté ^c^/IRB Yves Ponroy Canada Inc.	80021605	Soy isoflavones (300 mg)^c^	1, at night	$0.55
i-Cool®/i-Health, Inc.	80033608	Synthetic soy isoflavones, genistein (30 mg)	1, daily	$0.83-$0.87
A. Vogel™ Menopause/Bioforce Canada Inc.	80034857	Sage (3400 mg)	1, 1 to 5 times daily	$2.33-$2.50

Notes: ^a^products without a range of daily cost were either only found in a single pharmacy or found in several pharmacies at a single price.

^b^products share the same licence number.

^c^Menoconfort Forté packages include a daytime NHP for “radiant skin”.

**Table 2:** **Indications and contraindications/warnings for each product**

**Product**	**Indications**					**Contraindications/warnings**							
	**Menopause symptoms**	**Vasomotor**	**Sleep**	**Mood**	**Other**	**Pregnancy/breastfeeding**	**Hormonal gynecological disorders, cancer**	**Breast cancer**	**Liver disorder**	**Allergy to ingredients**	**Blood thinning medications**	**HRT/thyroid medications**	**Other**
**Products with multiple active ingredients**													
Swiss Natural HRT® Regular	+	+	-	-	-	+	+	+	+	+	+	+	
Swiss Natural HRT® Extra Strength	+	+	-	-	-	+	+	+	+	+	-	+	Diabetes
Swiss Natural HRT® Nightime	+	+	+	-	-	+	-	-	+	+	-	-	Avoid alcohol/sedatives
Estroven®	+	+	-	-	-	+	+	+	+	+	+	+	
Estroven® Maximum Strength	+	+	-	-	Bone health	+	+	+	+	+	-	+	
Life Brand™ Menopause Relief	+	-	-	-	-	+	-	-	+	+	-	-	Kidney stones
femMED® Menopause Relief	+	+	+	+	-	+	-	-	+	-	-	+	
FemmeCalm Menopause Formula	+	+	+	+	-	+	+	+	+	+	+	+	
MENOsmart™	+	+	-	-	-	+	-	-	-	-	+	-	
**Products with single active ingredient**													
up&up™ Black Cohosh	+	-	-	-		+	-	-	+	-	-	-	’
London *Naturals*® Black Cohosh	+	-	-	-		+	-	-	+	-	-	-	
equate® Black Cohosh	+	-	-	-		+	-	-	+	+	-	-	May cause drowsiness
Holista® Black Cohosh	+	-	-	-		+	-	-	+	-	-	-	
Be.better™ Black Cohosh	+	-	-	-		+	-	-	+	-	-	-	
Promensil® Regular Strength	+	+	-	-	Bone & heart health	+	+	+	+	+	+	+	
Promensil® Double Strength	+	+	+	+	Bone & heart health, vaginal dryness	+	+	+	+	+	+	+	
Soy Isoflavone Complex	+	+	-	-	Bone health	-	+	+	+	-	+	+	
Menoconfort Forté	+	+	-	-		-	+	+	+	-	+	+	
xi-Cool®	+	+	-	-		-	+	+	+	-	+	-	
A. Vogel™ Menopause	+	+	-	-		+	-	-	-	+	-	-	Epilepsy

### Regulatory approval

Nineteen of the products were labeled with an NPN (Table 1). One product had no available information regarding its regulatory approval. Three of the black cohosh products had the same NPN, indicating that each of these store “own brand” products were made under license by the same manufacturer, in this case WN Pharmaceuticals.

### Ingredients

A total of 28 different active ingredients were identified. The number of active ingredients per product varied from one to 19. Nine products contained multiple active ingredients, while eleven products contained a single active ingredient. The most common active ingredients were black cohosh (14 products), soy isoflavones (n = 7), chaste tree (n = 5), dong quai (n = 3) and red clover isoflavones (n = 3).

### Claims of efficacy and safety

In accordance with our product selection criteria every OTC product made a menopause symptom health claim. Fifteen of the 20 products asserted they were effective in alleviating “general menopausal symptoms” with or without specific symptom benefits. Fourteen of the 20 products claimed relief from vasomotor symptoms (Table 2), specifically hot flashes (n = 14) and night sweats (n = 10). One product claimed relief from urogenital symptoms, including vaginal dryness and low libido (Table 2). Four products claimed they would contribute to reduced bone loss if taken with sufficient Vitamin D and calcium (Table 2).

Each product had labeled contraindications for at least one specific condition. There was considerable overlap between contraindications reported, even though the active ingredients were different (Table 2). Eighteen of the NHPs warned against the use of the product with liver dysfunction and 17 warned against their use while pregnant or breastfeeding. Many of the products had a labeled contraindication involving the use of the product with blood thinning medications (n = 9) or hormone replacement therapy (n = 10). Other frequent contraindications included the use of the product with hormone-associated gynecological disorders such as endometriosis or cancers (n = 10) or history of or current breast cancer (n = 10). Warnings were usually about stopping taking the product in the event of new-onset liver symptoms or breast pain, or development of an allergy to any of the ingredients.

### Cost

The cost of the average recommended daily dose (CAD$) for each product varied widely, from a minimum of $0.07 to a maximum of $2.50 (Table 1). The cost of the recommended daily dose of single ingredient products on average (mean (SD) $0.71 (±$0.53)) was found to be identical to, but vary more than, the average daily cost of combination products ($0.71 (+$0.30)). Sale prices were responsible for some products being sold at bargain prices.

## Discussion

Our investigation revealed that Canadian pharmacy chains sold a variety of NHPs that claim they can provide relief from menopausal symptoms. Our study aimed to be as relevant as possible to the experience of women throughout Canada seeking over-the-counter assistance through the menopause transition. We explored the packaging information easily available to women making decisions in pharmacies about which menopause-related NHP to use. The descriptions of indications for use and contraindications are remarkably similar between the menopause treatments and therefore the cost of treatment becomes an important factor. According to our findings, the annual cost of purchasing OTC NHPs for menopausal symptoms can range from $29.20 to $872.35 (CAD$) for the average Canadian woman.

Natural health products available OTC for menopausal symptoms are assessed and licensed on the basis of safety and efficacy as per Health Canada’s regulation standards. However, while Health Canada requires evidence of the association of active ingredients with the health claim made by the product, the type and amount of evidence required depends directly on the health impact on consumers if the product does not work as expected. Menopause is considered a low-risk condition by Health Canada, in that symptoms are not life threatening and have the potential to resolve themselves over time [10], and Health Canada requires minimal efficacy evidence for natural health products in the low-risk category [11]. The Health Canada regulations stipulate that the details provided on the labels of licensed NHPs must include information on risks and possible interactions with conventional treatments. Furthermore, Health Canada requires that all NHP manufacturers have a site license, signifying that the site is able to maintain proper production, labeling, handling, and distribution of the products made there [18]. Health Canada rules on compliance with labeling [19] ensure that the proper protocol for labeling is being followed and the necessary information is being provided to consumers. However, the volume of mandatory information means that the labels use very small font sizes in order to provide the details in two languages, making the warnings inaccessible to anyone with poor eyesight or lack of understanding of the terms used. Potential purchasers might find information about warnings of possible risks unsettling [20] and seek additional information from pharmacists who must be prepared to answer detailed questions about the NHPs [21].

There have been many studies assessing NHP supplementation for relief of menopausal symptoms [for example 12–17]. However, the findings have been inconsistent, and the quality and heterogeneity of studies has reduced the number of trials that can be combined in systematic reviews or meta-analyses. An Australian systematic review carried out on OTC products for relief of hot flashes in menopausal women found contradictory research evidence of NHP supplementation’s effectiveness, concluding that the inconsistent data did not support the use of OTC products for menopausal symptoms [12].

Black cohosh has generated the most research interest as a treatment for menopause symptoms, of relevance to the results of our study which found that black cohosh was the most commonly available active ingredient, present in 70% of the products we identified. A 2012 Cochrane review of randomized controlled trials assessing black cohosh versus placebo or other treatments found that black cohosh did not significantly reduce hot flashes or menopausal symptom scores, whereas hormone treatment (compared to black cohosh) reduced symptom scores significantly [13]. A 2015 extensive review of effects of herbal preparations on symptom clusters in menopause suggested that black cohosh had a significant effect on hot flashes [22], but this observation was based on qualitative review of the research, rather than the more rigorous quantitative methods used in the Cochrane review of randomized trials [13].

Black cohosh has been suspected of being linked to liver disorders, although cases are rare and can seldom be attributed directly to black cohosh [14]. Evidence on safety of black cohosh from the Cochrane review was inconclusive because of inadequate reporting [13], and the more recent review suggested that the incidence of adverse effects associated with black cohosh did not differ from those associated with placebo [22].

A Cochrane review of the evidence supporting phytoestrogens (including soy and red clover isoflavones) for menopausal vasomotor symptoms produced no conclusive evidence that phytoestrogen supplementation effectively reduced the frequency or severity of hot flashes and night sweats [15]. A sub-group of trials comparing Promensil (red clover isoflavones) to placebo did not find a significant reduction in hot flashes [15]. A recent meta-analysis of the efficacy of phytoestrogens versus placebo indicated that hot flash frequency was reduced compared to placebo, but there was not a significant treatment effect of phytoestrogens on menopause symptoms more generally [16]. In the few studies that reported side effects, there was no significant difference between phytoestrogen and placebo [16]. A systematic review including chaste tree and black cohosh did not find either was more effective than the placebo in treating climacteric complaints [17]. Despite the lack of supporting evidence for the effectiveness of black cohosh, chaste tree or isoflavones in the management of menopause symptoms, the recently published North American Menopause Society “recommendations for the clinical care of midlife women” acknowledges that women often seek out this type of treatment [23].

It is worth noting that several studies on the efficacy of NHP supplementation mention a strong placebo response [13,24-28] ranging from 25% [24] to 63% [28]. Thus, it would seem that although the current literature does not show consistent efficacy evidence of the use of natural health products for menopause [12], women might still find relief of their symptoms from the placebo effect.

Our sample of OTC NHPs for menopause is a “snapshot” of products available in pharmacy chains across Canada. An apparent limitation to this study is that only nine Canadian pharmaceutical chains were investigated: between these chains, they are present in all provinces and territories in Canada and thus our sample represents a selection of the pharmacies where Canadian women would likely purchase an OTC supplement for menopausal symptoms. There are several other limitations of our study method. The stores we visited were in one city and we visited only one store per pharmacy chain. We cannot therefore comment on geographic or between-store variation. As well, availability does not necessarily reflect sales, although products that did not sell would be unlikely to remain for long on the store shelves. Our sample of NHPs represents the type and distribution of the 448 NHPs currently licensed by Health Canada for menopause, in terms of frequently found active ingredients and balance between single ingredient and multiple ingredient products [29]. For example, the following active ingredients are commonly in Health Canada licensed menopause NHPs: black cohosh (51%), chaste tree (36%), soy or red clover isoflavones (20%); and 45% of the licensed NHPs include a single active ingredient [29].

The objective of our study is limited to the investigation of OTC NHPs in Canada-wide pharmacies only. Nevertheless, apart from the store own-brand products (for example black cohosh), the products identified in this research are not restricted to the selected pharmacy chains and are likely available at independent pharmacies, naturopaths and herbalists. It is of note that many NHPs advertised for menopausal symptoms (including those identified in our study) can be bought outside of pharmacies, for example by mail order or online.

While this study is specific to the NHPs available in Canada, the use of NHPs in treating menopausal symptoms is not limited to Canada alone. Other investigations into the use of natural health therapies (black cohosh, isoflavones, phytoestrogens, and others) to treat the symptoms of menopause have been published throughout the world, including in the United Kingdom, Australia, Italy, and Turkey [30-33]. A brief informal search of pharmacy websites in Europe, Australia and USA found that Promensil® products and A.Vogel™ Sage, as well as many generic products of black cohosh, soy isoflavones, and chaste tree are widely available internationally. Therefore, our findings regarding the ingredients and doses of NHPs for menopause that are easily accessible to Canadian women have relevance beyond Canada.

Women are keen to make their own decisions about the use of NHPs in menopause [7], largely because of their concerns about perceived risks associated with hormone replacement therapy [4-6]. Women must balance the potential for benefit with the possible harm when making NHP decisions however the quality and type of evidence available to support women is often limited as illustrated by our study. It is important that women are aware that the regulation of these products does not imply their effectiveness, as the evidence required by Health Canada for licensing NHPs treating “low therapeutic impact conditions”, including menopause, reflects “the low risk nature of these products” [10,11]. Evidence-based decision aids designed to improve the quality of decision-making are fairly common to support menopausal women’s decisions about whether to use hormone replacement therapy [34]. Unfortunately few studies of decision aids regarding the use of NHPs have been undertaken [35,36].

Over-the-counter NHPs are widely available for women who wish to self-manage their menopausal symptoms. Future directions for research in this area may include investigation into the high placebo response seen in controlled trials of NHP supplements for menopausal symptoms, as well as examining the efficacy and safety of combination NHP supplements; most published scientific trials have solely investigated single-ingredient supplements, though almost half of the products identified in this study available for purchase were combination products. Adequately designed decision aids would incorporate the evidence that is available to support the use of NHPs as well as discussing the information that is unknown [34].

## Conclusion

Women seeking non-hormonal treatments for their menopause symptoms may prefer to use NHPs sometimes based on lay beliefs rather than evidence [37], incurring the potential of interaction with prescription medications [38,39]. Our study found that NHPs for menopausal symptoms are easily available in Canadian pharmacies. The lack of clear evidence of product efficacy, safety with respect to potential interactions with prescribed medications, and cost comparisons poses a challenge for women to make well-informed treatment choices. Further information is needed about the factors women take into account in making decisions about the purchase of NHPs.

## Abbreviations

NHP: Natural health product
NPN: Health Canada Natural Product Number
OTC: Over-the-counter
SD: Standard deviation
